# Multiple Sklerose und COVID-19

**DOI:** 10.1007/s00739-020-00691-z

**Published:** 2021-01-15

**Authors:** Michael Guger, Gerhard Traxler

**Affiliations:** Klinik für Neurologie 2, Kepler Universitätsklinikum GmbH, Krankenhausstraße 9, 4021 Linz, Österreich

**Keywords:** COVID-19, Multiple Sklerose, Krankheitsmodifizierende Therapien, Immunologie, SARS-CoV‑2, COVID-19, Multiple sclerosis, Disease-modifying therapies, Immunology, SARS-CoV‑2

## Abstract

Alter, Behinderungsgrad, Übergewicht und Komorbiditäten sind bekannte Risikofaktoren für den schweren Verlauf einer COVID-19-Erkrankung bei Patienten mit multipler Sklerose. Die Diagnose einer multiplen Sklerose per se stellt, nach aktuellem Wissensstand, jedoch keinen isolierten Faktor für eine Ansteckungsgefahr oder einen schweren Verlauf dar. Ebenso wird die Fortführung krankheitsmodifizierender Therapien aufgrund der bisherigen Erfahrungen als sicher und ohne generell erhöhtes Risiko für die Patienten erachtet. Eine bereits etablierte Therapie bei multipler Sklerose sollte daher auch trotz COVID-19-Pandemie fortgeführt werden. Bei Patienten mit entsprechender Indikation sollte die Einleitung einer Therapie nicht verzögert werden. Weitere Auswertungen zukünftiger Registerdaten zur Bestätigung der bisherigen Ergebnisse sind wünschenswert und notwendig.

## Abstract (en)

Age, higher EDSS (Expanded Disability Status Scale), obesity and comorbidities are known risk factors for severe course of COVID-19 in patients with multiple sclerosis. However, multiple sclerosis per se is not an isolated factor for higher risk of infection with SARS-CoV‑2 or severe course. So far disease-modifying therapy prevalence has not shown a difference in clinical outcome compared to patients without multiple sclerosis, but further confirmation is warranted. Treatment for multiple sclerosis should be continued despite the COVID-19 pandemic. In patients with appropriate indications, initiation of treatment should not be delayed. More analyses of future registry data to confirm the previous observations is desirable and necessary.

## COVID-19

Bis Ende November 2020 waren weltweit über 63 Mio. Menschen mit dem SARS-CoV‑2 infiziert, davon verliefen über 1,4 Mio. Infektionen tödlich.

Der klinische Verlauf ist durch drei mögliche Krankheitsphasen charakterisiert. In der frühen Infektionsphase weisen Patienten keine oder zumeist nur leichte unspezifische Beschwerden auf, wie Fatigue, Fieber und/oder trockenen Husten. Ebenso wird häufig über den Verlust des Geruchs- und Geschmackssinns berichtet. Etwa die Hälfte aller Infektionen ist nach Phase eins beendet. Die andere Hälfte bildet in Phase zwei unterschiedlich deutliche Krankheitssymptome bis hin zu einer ausgeprägten viralen Pneumonie aus. Bei anhaltender unkontrollierter Infektion kommt es in Phase drei zu einer systemischen Hyperinflammation und dem sog. „Zytokinsturm“ mit entsprechend schlechter Prognose.

Identifizierte Risikofaktoren für einen schwereren Verlauf in der Allgemeinbevölkerung sind höheres Alter, Übergewicht und Komorbiditäten, wie zum Beispiel kardiovaskuläre Erkrankungen, chronische Lungenerkrankungen und Diabetes mellitus Typ 2.

Risikofaktoren sind Alter, Behinderungsgrad, Übergewicht und Komorbiditäten

Das Virus SARS-CoV‑2 wird hauptsächlich per os über Tröpfcheninfektion und Aerosole übertragen. Es dringt über eine Bindung an das in der Zellmembran verankerte Enzym ACE2 in die menschliche Zelle ein. Hierbei interagiert das virale Spikeprotein mit ACE2. Für diesen Prozess ist die Mitwirkung der Serinprotease TMPRSS2 notwendig. Es erfolgt zunächst die Hochregulierung einer Typ-I-Interferon-Antwort durch direkte Freisetzung des spezifischen Transkriptionsfaktors NF-κB („nuclear factor kappa-light-chain-enhancer of activated B‑cells“). Hierdurch kommt es zur Aktivierung von Signalwegen des angeborenen Immunsystems. Es werden verschiedene proinflammatorische Zytokine, wie zum Beispiel IL-1β, IL-1Ra, IL‑6, IL‑7, IL-10, IP-10 und TNFα, hochreguliert, welche letztlich die systemische Inflammation weiter bis zum unkontrollierten „Zytokinsturm“ vorantreiben können. Dementsprechend ist ein erhöhter IL-6-Serumspiegel mit einem schlechteren klinischen Ergebnis verbunden. Das adaptive zelluläre Immunsystem, allen voran CD4-zentrale-Gedächtnis- und CD8-Effektor-Gedächtniszellen, spielen eine fundamentale Rolle in der Bekämpfung der SARS-CoV-2-Infektion. Im Labor sind eine milde bis schwere Lymphopenie, vor allem von T‑Lymphozyten und natürlichen Killerzellen, und eine Erhöhung der Neutrophilen zu beobachten. In Bezug auf die humorale Immunantwort ist die Serokonversion von neutralisierenden IgG-Antikörpern im Schnitt 7–14 Tage nach Symptombeginn zu beobachten. Bei symptomfreien oder milden klinischen Verläufen wird jedoch oftmals ein rascher Abfall des Titers beobachtet. In einem hohen Prozentsatz weisen diese Patienten nach Monaten bereits wieder eine Seronegativität auf.

## COVID-19 und multiple Sklerose

Entsprechend der hohen Prävalenzzahlen gibt es weltweit viele Multiple-Sklerose(MS)-Patienten mit einer bereits durchgemachten COVID-19-Erkrankung. Durch Dokumentation und Auswertung der Fälle in verschiedenen nationalen MS-Registern konnten Vergleiche zur Normalbevölkerung erstellt werden und Risikofaktoren für einen schweren Krankheitsverlauf identifiziert werden.

In der italienischen Auswertung von 232 COVID-19/MS-Patienten wies der Großteil (*n* = 222, 95 %) einen milden Verlauf auf. In zehn Krankheitsfällen wurde der Verlauf als schwer und/oder kritisch beschrieben. Fünf Patienten sind an den Folgen der Infektion verstorben.

Ähnliche Erkenntnisse wurden aus dem französischen Register gewonnen. Von 347 dokumentierten an COVID-19 erkrankten MS-Patienten mussten 73 im Krankenhaus behandelt werden. 12 Erkrankte (3,5 %) verstarben an den Folgen der SARS-CoV-2-Infektion. Eine MS-spezifische Behandlung, im Sinne von Immunmodulation oder Immunsuppression, stellte hierbei keinen Risikofaktor im Vergleich zu unbehandelten Patienten oder zur Allgemeinbevölkerung dar. Unabhängige Risikofaktoren für einen schweren Krankheitsverlauf waren höheres Alter, höherer EDSS und Übergewicht.

In einer amerikanischen Observation zeigten 6 von 40 mit COVID-19 erkrankten MS-Patienten (15 %) einen schweren Verlauf und die Notwendigkeit einer intensivmedizinischen Versorgung. Als Risikofaktoren für einen unvorteilhaften Verlauf konnten wiederum höheres Alter sowie ein chronisch progredienter MS-Verlauf identifiziert werden. Das Vorhandensein einer Immunmodulation bzw. Immunsuppression hatte keinen nachweisbaren Einfluss auf die Schwere des Verlaufs.

## COVID-19, multiple Sklerose und Schwangerschaft

Grundsätzlich erscheint das Risiko im Hinblick auf den Schweregrad einer COVID-19-Erkrankung von Schwangeren nicht erhöht. Ebenso kann kein Unterschied in der Mortalität im Vergleich zur Normalbevölkerung festgestellt werden. Es gibt jedoch Hinweise, dass trotzdem die Häufigkeit der Notwendigkeit einer intensivmedizinischen Behandlung erhöht sein könnte. Zudem weisen die bisher publizierten Studien auf eine möglicherweise erhöhte Frühgeburtenrate von mit COVID-19 erkrankten schwangeren MS-Patientinnen hin. Letztendlich wird ein stringentes „social distancing“ ab der 28. Schwangerschaftswoche empfohlen, ohne jedoch mögliche Folgen einer sozialen Isolation außer Acht zu lassen.

## COVID-19 und MS-spezifische Medikamente

In mehreren Studien konnte bereits ein antiviraler Effekt (einschließlich auf Coronavirusinfektionen) von Interferon‑β nachgewiesen werden. Dementsprechend wird ein möglicher protektiver Effekt unter dieser Therapie diskutiert.

Ebenso besteht bei Teriflunomid und dessen Pro-Drug Leflunomid ein potenzieller antiviraler Wirkmechanismus. Leflunomid wurde experimentell bereits bei COVID-19 als antivirale Therapie eingesetzt. Bisherige Fallstudien konnten kein erhöhtes Risiko für einen schweren Verlauf einer SARS-CoV-2-Infektion unter Teriflunomid nachweisen.

Daten zu Glatirameracetat und einer COVID-19-Erkrankung weisen bisher ebenso keine negativen Auswirkungen auf den Krankheitsverlauf auf.

Für Dimethylfumarat (DMF) wurde bis dato kein erhöhtes Risiko eines schweren Verlaufs bei COVID-19 beschrieben. Zu beachten ist, dass DMF einerseits über die Aktivierung des Nrf2 („nuclear factor erythroid 2-related factor 2“) Transkriptionsfaktors wirkt, welcher in vitro durch SARS-CoV‑2 inhibiert wird und somit auch die Virusreplikation beeinträchtigen könnte. Andererseits kann durch DMF, vor allem bei älteren Patienten, eine höhergradige Lymphopenie hervorgerufen werden, die das Infektionsrisiko potenziell erhöht.

MS-Patienten unter einer Therapie mit Sphingosin-1-Phosphat(S1P)-Modulatoren wiesen zu Beginn der COVID-19-Erkrankung oftmalig eine höhergradige Lymphopenie auf. Trotzdem kam es zumeist zu einem mild bis moderaten Krankheitsverlauf. Laut Studien zum „acute respiratory distress syndrome“ (ARDS) könnte eine Therapie mit S1P-Modulatoren einen positiven Einfluss auf die Lungenschädigung bewirken. Im Gegensatz dazu wurde in Phase-III-Studien bei MS eine erhöhte Rate an unteren Atemwegsinfektionen und Herpesinfektionen nachgewiesen.

Bisherige Daten zu COVID-19 und Cladribin zeigten kein erhöhtes Risiko für einen schweren Verlauf. Eine signifikante Erhöhung von viralen Infektionen (bis auf Herpes zoster) konnte unter Cladribin gegenüber der Placebobehandlung nicht nachgewiesen werden, obwohl unter Therapie mit Cladribin in seltenen Fällen eine Lymphopenie IV° mit erhöhter Infektanfälligkeit auftreten kann.

Durch die Blockade von α4-Integrin verhindert Natalizumab den Übertritt von Lymphozyten über die Blut-Hirn-Schranke. Durch die verminderte Immunüberwachung im zentralen Nervensystem werden potenzielle neurologische Komplikationen bei einer SARS-CoV-2-Infektion unter Natalizumab diskutiert. Bisherige Fallberichte zeigten keine gehäuften schweren Verläufe.

Anti-CD20-Antikörper weisen durch die B‑Zell-Depletion und die nachfolgende reduzierte T‑Zell-Antwort ein möglicherweise erhöhtes Infektionsrisiko bei multipler Sklerose auf. Verschiedene internationale Kohortenstudien suspizierten ein erhöhtes Risiko für einen unvorteilhaften Verlauf unter Ocrelizumab bzw. Rituximab. Postuliert wird zudem eine potenziell erleichterte Reinfektion mit dem SARS-CoV-2-Virus bei verminderter Antikörperproduktion gegen COVID-19 unter besagten Therapien.

Alemtuzumab wies in gepoolten Studiendaten bei MS eine erhöhte Infektionsrate vor allem in den ersten beiden Jahren nach Therapiebeginn auf. Die Repopulation von natürlichen Killerzellen verläuft jedoch zumeist rascher als unter einer Cladribintherapie. Bisherige Erfahrungen von COVID-19 und Alemtuzumab wiesen einen überwiegend milden Krankheitsverlauf auf.

Die MS-Schubtherapie durch hoch dosiertes Methylprednisolon, vor einer COVID-19-Erkrankung verabreichtet, wies ein erhöhtes Risiko für schwere Krankheitsverläufe auf. Beim schweren Krankheitsverlauf einer SARS-CoV-2-Infektion hingegen kann eine Kortisontherapie den gefürchteten Zytokinsturm unterdrücken.

MS-Therapien weisen kein generell erhöhtes Risiko auf

Bei allen MS-Patienten mit immunmodulatorischer oder immunsuppressiver Behandlungsindikation sollte es zu keiner Verzögerung des Therapiestarts aufgrund der COVID-19-Pandemie kommen. Dem potenziellen Infektionsrisiko einer SARS-CoV-2-Infektion muss das Risiko der Krankheitsaktivität durch verzögerten Therapiestart gegenübergestellt werden. Die soeben genannten Erfahrungen und Überlegungen sollten daher bei der Wahl der Therapie berücksichtigt werden.

Ebenso kann bei eindeutigem Schubgeschehen und entsprechender Klinik ein Kortisonstoß nach individueller Nutzen-Risiko-Abwägung durchgeführt werden.

Eine klare Empfehlung für das Vorgehen bei Infektion mit SARS-CoV‑2 unter immunmodulierender oder immunsuppressiver Therapie hinsichtlich Fortführung der Therapie gibt es nicht. Depletierende Therapien dürfen bei jeglichem systemischen Infekt nicht verabreicht werden und sollten frühestens nach Infektausheilung nachgeholt werden. Schwieriger ist das Vorgehen bei anderen verlaufsmodifizierenden Therapien, da einerseits der Krankheitsverlauf zum Infektionsbeginn nicht vorhersehbar ist und andererseits die jeweilige Therapie über die Einnahme anhaltende Effekte aufweist. Zudem besteht vor allem bei Therapien der hochaktiven MS die Gefahr des Rebound-Phänomens unter Therapiepause. Prinzipiell sollte, sofern vertretbar, die verlaufsmodifizierende Therapie unverändert fortgeführt werden. Gegebenenfalls kann auch ein „extended intervall dosing“ zur Vermeidung von Rebound-Effekten in Betracht gezogen werden. In allen Fällen sollten die Patienten über die unzureichende Evidenz aufgeklärt und in die Entscheidung miteingebunden werden.

Bestehende Therapien sollten fortgeführt werden

Zusammenfassend wird der Verlauf einer COVID-Erkrankung bei MS-Patienten durch dieselben Risikofaktoren wie in der Normalbevölkerung bestimmt (Tab. 1). Die Krankheit multiple Sklerose an sich stellt hierbei kein erhöhtes Risiko dar. Jedoch gilt die präexistente Behinderungsprogression gemessen am EDSS als beachtenswerter isolierter Risikofaktor. Je nach MS-spezifischer Immunmodulation und/oder Immunsuppression sind keine oder besondere Vorsichtsmaßnahmen zu beachten (Tab. 2).

**Table 1:** 

Risikofaktor	Niedriges Risiko	Intermediäres Risiko	Hohes Risiko
*Alter*	<45	45–60	60
*Begleiterkrankung(en)* (z. B. kardiovaskuläre, pulmonale oder Tumorerkrankungen)	Keine	Eine	Mehrere
*Übergewicht* (Body-Mass-Index)	Kein oder geringes Übergewicht (18,5–29,9)	Moderates Übergewicht (30–40)	Starkes Übergewicht (>40)
*Rauchen*	Nein	Ja	Ja
*EDSS*	0–4	4–6	>6
*DMT-assoziierte Lymphopenie*	Grad 0–1	Grad 2	Grad 3–4

*EDSS* Expanded Disability Status Scale, *DMT* Disease Modifying Therapies

**Table 2:** 

	Alemtuzumab	Cladribin	Dimethylfumarat	Fingolimod	Glatirameroide	Interferon‑β	Mitoxantron	Natalizumab	Ocrelizumab	Rituximab (off-label)	Teriflunomid
(Potenzielle) immunsuppressive Wirkung	Ja	Ja	(Ja)	Ja	Nein	Nein	Ja	(Ja)	Ja	Ja	(Ja)
Laborkontrollen (z. B. Blutbild, Diff.-BB)^a^	●	●	●	●	–	●	●	●	●	●	●
*Beginn* DMT in der derzeitigen Situation	(✓)^b^	(✓)	✓	✓	✓	✓	✗	✓	(✓)	(✓)	✓
Reguläre *Weiterführung* DMT in der derzeitigen Situation	(✓)^b^	✓	✓	✓	✓	✓	✗	✓^c^	✓	✓	✓

*Diff.-BB* Differenzialblutbild, *DMT* Disease Modifying Therapies

^a^Frequenz von der jeweiligen Fachinformation abhängig

^b^Unter Abwägung eines potenziellen Infektionsrisikos in den ersten 3–4 Monaten nach jedem Therapiezyklus

^c^Extension des Verabreichungsintervalls auf 6 Wochen kann aus logistischen Gründen (Reduktion der Infusionsfrequenz) erwogen werden

## Fazit für die Praxis

- Risikofaktoren für einen schweren Verlauf von COVID-19 bei MS sind Alter, Behinderungsgrad, Übergewicht und Komorbiditäten.
- Krankheitsmodifizierende Therapien weisen kein generell erhöhtes Risiko auf.
- Bestehende Therapien sollten trotz COVID-19-Pandemie fortgeführt werden.
- Vorsicht vor verzögertem Therapiebeginn und/oder Auswahl einer weniger wirksamen Therapie.

